# Woman’s Dress a Cause of Uterine Displacements

**Published:** 1851-10

**Authors:** 


*Woman's Dress a Cause of Uterine Displacements.* Read before the Boston Society for Medical Improvement, July 28th, 1851, by Dr. W. E. Coale.

The great and increased frequency of uterine displacements in the last few years, must have forced itself upon the attention of every practitioner of medicine. A peculiarity, too, that they have of late assumed is, that they are now met with in very young persons, whilst medical authors, writing not a quarter of a century ago, describe them, unless in exceptional cases, as affections to be found in women who have several times undergone the labors of a mother — in those of originally defective constitutions — in those who have been imprudent in making exertions too soon after childbirth — or, in short, in those who have been worn down and enfeebled by any cause calculated to lessen the general tone of the system: imprudence in habits of life — overtasking in particular occupations requiring a stooping position, decay from age, &c. We find, however, now — and I appeal to those present for a candid confirmation or contradiction of the assertion — that a large number of cases of prolapsus uteri occurs in those in early womanhood, and some in those who have scarcely advanced beyond girlhood. For my own part, without recurring to former cases, the fact that at this moment I have under my care five — not one older than twenty-three, one of them but eighteen years of age, not one of them a mother, none engaged in any exhausting occupation — gives me warrant for what I say, and, though accident may just now have greatly increased my proportion of such cases, I cannot believe that in the total my experience is very different from that of others present. It is, then, surely an interesting subject for inquiry as to what are the causes of the frequency of these affections just now; and why are the youngest, and, in other respects, the heartiest women the victims of it?

One undoubted explanation for some of this frequency is, that from an increase of medical research and inquiry upon the subject, the disease is now detected where formerly it was passed by unrecognized, so that the increase of frequency is not so great as at first might be imagined. I state this in the outset plainly, that it may have its full force as far as it can go, and that it may not be supposed that I have at once gone to a favorite theory, not looking carefully and without prejudice to other sources.

Throwing out, then, a fair proportion of cases, as accounted for above, we still have left a large number for which we must seek other means of accounting. These, we believe, we find in the mode of dress now in fashion among our women — the peculiarity of which, as interesting to us is, that it is supported almost entirely from the waist — using that word, not in the dressmaker's sense, but in its old meaning as designating the contracted portion of the figure just above the hips.

Until the last fifteen years, although the dress was at times worn very low on the chest, it was always hung by broad shoulder-straps, frequently coming from the shoulders very high up toward the sides of the neck. A reference to any prints illustrating the fashions of this century prior to the time mentioned, or the costumes of England or France for any period, will more fully explain this if necessary. About fifteen years since, as a ball-dress, the shoulder straps were left off, so that the upper line of the dress was perfectly horizontal, and this, with those elastic views of delicacy so peculiar to fashion, was often low enough to disclose the edge of the armpit. In this style there was apparently great danger of the dress slipping down, and it would do so but for the ingenious, though not graceful, contrivance of suspending it from uprights of whalebone, the lower ends of which are supported at the waist. This, from being a ball costume, has become more and more common; so that now, even when high-necked outer dresses are worn, the under dresses are cut low and supported as above described, in order to suit if a change be made in the former. Thus much for the part of the dress above the waist, to which we attribute its measure, though not a very large one, of the affections under consideration.

To the part below the waist, however, we believe we can look with confidence for a full and satisfactory explanation of the mischief done.

With a view of improving their shape, the lower part of the dress of women now consists of six, eight, or even more, skirts,* made of various materials; cotton — the stiff woollen material, intended for curtains, called moreen — flannel, and at times, quilted with cotton wool, weighing together, as ascertained by actual experiment, ten, twelve, and even fifteen pounds. Each of these is supported by a string drawn very tightly around the body. We have seen the marks of these strings for days after the skirts have been removed —

* This is on the confession of patients themselves, or I could not believe or dare state it.

we have seen them even after death. Here, then, is the first source of evil — the continued pressure and constraint that these strings keep up — evidently embarrassing greatly the organs within. When to this, however, we add the weight of the skirts, we cannot but at once perceive how great an additional force we set to work, particularly if its operation — as exerted upon organs having among themselves a mobility almost as great as that of fluid — be properly estimated. To protect the abdominal viscera against this pressure, remember there is nothing, in front, at least, save a thin partition of woman's soft and tensionless muscle. That these viscera should be forced downwards is not surprising; that they must, in turn, exert an equal force downward on the pelvic viscera, is apparent; and that the uterus, the most movable of the last, and the most obnoxious by its situation to receive such an impulse, should give way to the continual assaults upon it, is what we might most readily expect from the premises. Here we have an explanation full, and, we trust, convincing, of the frequency of a disease in the youngest and heartiest of the sex, which, twenty years since, was considered peculiar to those whose powers of life were greatly exhausted by demands upon them, or were already on the decline from age, an explanation, I may mention in passing, not yet offered, as far as I can ascertain, by any other writer.

We look upon the mischief thus done as no whit less than that effected by tight lacing; but, if anything, greater, for it is more silently done. Friends cannot see, and do not understand, the evil at work, and, therefore, can give no warning word. The symptoms themselves commence so gradually, and point so indirectly to the cause, as to excite no alarm in the victim. Exercise, which ought to invigorate, soon fatigues and becomes distasteful. Ascending a flight of stairs, or stooping to lift a comparatively light weight, instantly loads the hips with a burden that can scarcely be borne. The back, particularly at the lower part, feels sprained, and memory is taxed in vain for some injury to account for it. Dragging sensations around the hips, pain down the legs, and weak knees, are attributed to rheumatism. The symptoms may now begin to point more directly to the real seat of the trouble — every monthly period brings renewed sufferings, from which the system rallies more and more slowly —daily and hourly embarrassments occur of nearly all the organs within the pelvis — an irritable bladder — (a very frequent symptom in my experience) — hæmorrhoids — unceasing pain and continual sensation of bearing down. The retiring delicacy of maidenhood shrinks from telling these, and unless marriage happily brings her under the care of a physician, the mischief goes beyond the hope of relief.

Displacement of the uterus, though the most permanent and grievous trouble produced by the heavy skirts, is not the sole one. Close observation and more particular inquiries into the symptoms of dysmenorrhœa, have convinced me that in very many cases the pressure above described keeps up, if it does not actually induce, a plethora of that organ, to which much of the sufferings at those periods may reasonably be attributed. This plethora, too, cannot be repeated often, or continued for a great while, it is evident, without alterations in the uterus itself, which must tend still further to embarrass it in the performance of its functions, and entail suffering upon the patient. Acting upon my conviction of this cause of suffering at the monthly periods, I have advised, upon the first warning of the flow commencing, that the string around the waist should be loosened, and as many of the skirts removed as the temperature will permit; and this I have often found to give immediate relief to a great degree.

If my theory as to the cause of so many of the cases of uterine displacement be correct, we have with it an explanation also of the inefficiency of our means of remedying the disease. Any truss or abdominal supporter, to be efficient, acting precisely as the skirts do, by pressure externally upon the walls of the abdomen, must exercise a pressure fully equal to them before it can begin to do any thing towards supporting the uterus. This is too clear to require demonstration. If it does act with equal force, we ask what can be the situation of a woman with a twelve-pound force pressing downwards and a twelve-pound force pressing upwards, upon the soft walls of the abdomen? What chance have the organs within of doing their duty, and how long, under such treatment, will it be before she can expect to lay aside such aids and assistances and find herself a well and hearty woman, with the original complaint perfectly remedied?

As a palliative to the evil of wearing such oppressive garments, we always recommend that they should be supported by shoulder-straps; and the suggestion of this simple expedient, imperfect as it is, has of itself brought us the heartiest thanks of the sufferers for the relief it has given them, assuring us that were the improvement carried further, in lighter and more equally-supported garments, greater relief might be afforded to our patients; and many, who are not such now, might be saved from becoming invalids.

The importance of the subject, I trust, will be a sufficient apology for the length of this paper, which I have tried to make as concise as clearness will permit. With a view to this, I have omitted to relate particular cases, though I could give several, highly illustrative of the correctness of my views, as well as more especial confirmations from expressions of patients themselves, often clothed in the strongest language that relief from suffering and renewed health uses.

In conclusion, I call attention to a moral aspect of the subject, viz.: that of all the peculiarities of woman's dress, which an appeal to the laws of physiology shows conclusively must seriously influence her health — low-necked dresses, corsets, tight and constraining waists, heavy skirts, narrow and thin-soled shoes — for not one of them is the shadow of a claim made that they contribute in the slightest to ease and comfort; but, on the contrary, it is openly professed that they are used solely and entirely for the improvement of the figure. By which we are driven to the inevitable conclusion that either woman was sent "into this breathing world scarcely half made up," or that French dressmakers have greatly improved upon the pattern as originally devised by the Creator.—*Boston Medical Journal.*

